# Disturbance Driven Colony Fragmentation as a Driver of a Coral Disease Outbreak

**DOI:** 10.1371/journal.pone.0057164

**Published:** 2013-02-20

**Authors:** Marilyn E. Brandt, Tyler B. Smith, Adrienne M. S. Correa, Rebecca Vega-Thurber

**Affiliations:** 1 Center for Marine and Environmental Studies, University of the Virgin Islands, Saint Thomas, United States Virgin Islands, United States of America; 2 Department of Microbiology, Oregon State University, Corvallis, Oregon, United States of America; 3 Department of Biological Sciences, Florida International University, North Miami, Florida, United States of America; 4 Department of Ecology and Evolutionary Biology, Rice University, Houston, Texas, United States of America; University of New South Wales, Australia

## Abstract

In September of 2010, Brewer's Bay reef, located in St. Thomas (U.S. Virgin Islands), was simultaneously affected by abnormally high temperatures and the passage of a hurricane that resulted in the mass bleaching and fragmentation of its coral community. An outbreak of a rapid tissue loss disease among coral colonies was associated with these two disturbances. Gross lesion signs and lesion progression rates indicated that the disease was most similar to the Caribbean coral disease white plague type 1. Experiments indicated that the disease was transmissible through direct contact between colonies, and five-meter radial transects showed a clustered spatial distribution of disease, with diseased colonies being concentrated within the first meter of other diseased colonies. Disease prevalence and the extent to which colonies were bleached were both significantly higher on unattached colony fragments than on attached colonies, and disease occurred primarily on fragments found in direct contact with sediment. In contrast to other recent studies, disease presence was not related to the extent of bleaching on colonies. The results of this study suggest that colony fragmentation and contact with sediment played primary roles in the initial appearance of disease, but that the disease was capable of spreading among colonies, which suggests secondary transmission is possible through some other, unidentified mechanism.

## Introduction

Multiple disturbances, including disease, have resulted in a steady and significant decline in the abundance of reef-building corals throughout the Caribbean [1]. In some cases, Caribbean reefs have shifted from coral-dominated states to algae-dominated states [2], [3], [4]. While there is evidence for resilience on some reefs [5], many projections for coral reefs under current disturbance regimes are grim [6], and our understanding of how disease affects the resilience of reefs is limited. Due to the potential of disease to cause extensive mortality within populations, there is a definitive need to understand the factors that influence the initiation and progression of disease outbreaks in corals.

Diseases that cause rapid tissue loss in Caribbean corals are collectively referred to as white diseases [7], and have been identified as primary contributors to recent declines in shallow water coral cover [8]. These diseases include three types of white plague disease (referred to as type 1, 2 or 3) that affect multiple species of reef-building corals [9], however the identification and etiological origins of white plague disease types remain highly controversial [10]. Characteristic signs of these diseases include lesions that originate at the base or margin of a coral colony and progress rapidly, resulting in large areas of denuded skeleton that appear bright white [7]. In “bleached” coral tissues, bright white skeleton is visible underneath transparent or lightly pigmented coral animal tissue, due to the loss or degradation of brown endosymbiotic microalgae that typically live in the coral's endodermis [11]. While coral bleaching does not always result in the animal's death, since living tissue that is bleached can be repopulated by pigmented algae [12], new white plague lesions, which are visually reminiscent of bleaching, contain no remaining tissue. Therefore lesions and bleached areas are distinguishable based on the absence of coral tissue in lesions and the presence of coral tissue in bleached areas.

White plague disease types are distinguished from one another primarily based on rate of linear lesion progression; white plague type 1 progresses the slowest (max 3 mm day^−1^) [13] and types 2 and 3 progress more rapidly (max 2 cm day^−1^ or greater, respectively) [14], [15]. No etiological agent has been demonstrated for types 1 or 3, but a novel bacterium, *Aurantimonas coralicida*, was identified for white plague type 2 [16]. However, in other studies, this pathogen was not detected from lesions with similar or identical descriptions to white plague type 2 [17]. This suggests that multiple etiologies may result in similar gross disease signs [10], which may or may not be infectious [18], but inconsistencies in the reporting of epidemiological characteristics of disease signs also may have contributed to conflicting results among studies [19]. Nonetheless, since it is clear that white plague-like diseases are significantly impacting Caribbean reef communities [20], thorough characterizations of their epidemiological properties should continue to be pursued because these efforts could identify important processes driving disease incidence. It is vital that detailed information be reported on the epidemiological properties of these diseases in order to minimize confusion among current and future etiological studies and to provide a basis for understanding disease incidence and spread.

In the fall of 2010, a localized outbreak of a white plague-like rapid tissue loss disease was identified in Brewer's Bay, St. Thomas Island, U.S. Virgin Islands (USVI). This outbreak occurred in conjunction with mass bleaching due to high temperatures and the passage of Hurricane Earl, a category 2 storm. Gross descriptions of the disease lesions matched closely with previous descriptions of white plague types 1, 2 and 3; lesions initiated basally or peripherally and progressed rapidly with a distinct margin separating living tissue from recently denuded skeleton [13], [14], [15], [16]. Here, we describe important epidemiological characteristics of the disease, including its spatial distribution, species affected and lesion progression rates. We compare these characteristics with those previously described for the three Caribbean white plague disease types in order to determine whether the disease was likely one of the three previously described types. Coral responses documented here were most similar to white plague type 1, but in order to avoid confusion until an etiology can be confirmed, we have followed the conventions suggested by Work and Aeby [19], and refer to this disease hereafter as the Virgin Islands multispecies rapid tissue loss disease (VI-MRTL).

The documented outbreak of the white plague-like VI-MRTL corresponded with two other significant disturbances: mass bleaching due to accumulated thermal stress and the passage of a category 2 hurricane that resulted in damage to and fragmentation of coral colonies. Outbreaks of white diseases in Caribbean corals have previously been documented in association with anomalous thermal events [21], [22] and storm disturbance [23]. White plague has been determined to occur on more extensively bleached corals during mass bleaching events [24], and colony breakage may play an important role in the occurrence of disease [25]. Mass bleaching events and storms, both dependent on warm sea temperatures, are expected to occur more frequently and at greater extremes in the Caribbean region as sea water temperatures rise [26]. While the cooling influence of hurricane passage has been shown to alleviate bleaching in some cases [27], physical impacts to corals and the incidence of disease can still result in significant mortality. The simultaneous occurrence of the disease outbreak with two other disturbances in this study allowed for an investigation into potential synergisms among disease, bleaching and damage from storms, which no study, to our knowledge, has investigated. We hypothesized that disease would occur more frequently on damaged corals and that diseased and damaged corals would be more extensively bleached.

## Materials and Methods

### Study site

The study site was located on Brewer's Bay reef, a nearshore fringing reef located in an embayment on the south side of St. Thomas Island in the U.S. Virgin Islands (GPS coordinates of the site: 18° 20′ 38.9, −64° 58′ 56.6). This reef is located 0.5 km from shore and occupies an area of approximately 0.08 km^2^. Consolidated reef development within Brewer's Bay occurs along a shallow slope between depths of 5 and 15 m.

### Field data collection methods

#### Population assessment

Transects were used to perform coral health assessments in Brewer's Bay during and after the event. Sites were assessed approximately every other week during the peak of the thermal stress (25 Sept, 11 Oct, and 22 Oct 2010), and then on two subsequent occasions after thermal stress had declined (23 Nov 2010 and 6 Feb 2011). At least three randomly located and oriented 10×1 m belt transects were used to quantify health status parameters of colonies of *Montastraea annularis*. Transects all were conducted at between 5–7 m depth and initially located using a list of random compass headings and distances. Only colonies with at least 50% of their skeletal structure within the belt transect were assessed, and the maximum diameter of these colonies was recorded to the nearest 5 cm.

In order to assess the health of each colony, the following parameters were recorded: 1) presence of VI-MRTL lesions, 2) presence and extent of colony bleaching, 3) evidence of physical colony damage (e.g., fragmentation), and 4) the presence of suspected disease vectors. 1) The presence of the disease or VI-MRTL lesion was defined as an area of denuded skeleton appearing bright white with little to no algal colonization that was located at the base or margin of the coral colony. It also was noted if the colony exhibited one or more disease lesions. 2) The presence of bleaching was defined as the absence of observable pigmentation in living coral tissue, while bleaching extent was measured as the % of a colony's living tissue area affected. 3) Physical colony damage included indications of storm-related damage, such as scouring and/or fragmentation. If the colony was noted as a fragment (i.e., not attached to the substrate), the substrate on which the fragment was laying was noted as either unconsolidated (sand or silt) or consolidated (hard) substrate. 4) The presence of potential coral disease animal vectors (i.e., corallivorous snails, fireworms, damselfish) was noted only if vectors were directly on the colony or, in the case of damselfish, were engaged in behaviors directly associated with the colony (e.g., guarding a nearby garden). Any contact between living tissue and various macroalgal species was noted.

#### Spatial data

The spatial distribution of diseased colonies was quantified using radial transects on 2 October 2012. Randomly selected diseased (test) and unaffected (control) colonies served as the center of spatial radial transects. An “unaffected” colony was defined as a colony with no observable active disease lesions. To initiate these transects, observers used a table of random compass directions and distances to navigate to an area of the reef. The observer then alternately selected the closest diseased or unaffected colony of *Montastraea annularis* as the center of a circular 5 m radial transect. Any diseased colony, regardless of species, within a 5 m radius of the selected central colony was assessed for its health status and the distance between its center and that of the central colony was measured. All other colonies within the radial transect were not assessed because of diving time limits, but the distribution of coral colonies and *M. annularis* at Brewer's Bay was observed to be fairly even.

#### Lesion progression rate calculation

In order to quantify lesion progression rates, four colonies were photographed on 23 September 2010 and then re-photographed from the same angle on 25 September 2010. A plastic ruler was placed in the frame of each photograph for scale. In the first photograph taken for each colony, four measurements of the linear distance between the line demarcating living tissue from recently denuded skeleton and an identifiable stationary point were made. The same procedure was then performed on the second photograph of each colony. The linear rate of lesion advance per day (cm/day) was calculated as the difference in the distance (cm) measured from the two photographs divided by the number of days between photographs. All measurements were made using the image analysis software Image J (NIH).

#### Transmission experiments

A direct transmission experiment was set up to determine whether disease signs were transmissible between colonies. This experiment tested whether direct contact between a disease lesion and apparently healthy tissue at the edge of an unaffected colony could result in disease signs. On 23 September 2010, individual diseased fragments or diseased colonies that were structurally intact where no breakage was noted (henceforth referred to as “attached”) were connected to individual unaffected attached colonies or fragments (n  =  20 total diseased-unaffected interactions) using new plastic cable ties. Specifically, 15 diseased fragments were paired with 15 unaffected attached colonies, and 5 diseased attached colonies were paired with 5 unaffected fragments. To distinguish between the appearance of disease lesions due to direct contact with a diseased coral and lesions that may have appeared due to the interaction or competition between two colonies, another 20 pairs of unaffected fragments and unaffected attached colonies served as controls. Pairs were observed approximately weekly for signs of disease transmission. The experiment was ended 1 December 2010, which constituted a total of 10 weeks of observations. All diseased and unaffected fragments were collected from reef substrate and sand at least 20 m away from the attached paired colonies. No fragment-fragment pairings were made due to the potential for them to be lost with wave action. Unmanipulated control colonies were not included in this experiment, but no new disease signs appeared on the attached experimental corals outside of those stimulated by direct contact.

#### Video records of lesions

An underwater video camera was installed *in situ* at Brewer's Bay to monitor the progression of lesions over 22 hours in order to determine whether predation partially or wholly contributed to lesion progression. A colony with two lesions was selected for video monitoring based on its amenability to filming (i.e., position of lesions on colony and the ability to install the camera securely near the colony). The camera was made negatively buoyant with lead weights and mounted on the seafloor 0.5 m from the colony. Two Princeton Tec Shockwave LED underwater dive lights that provided a maximum of 20 hours of light were attached to stakes and were placed so that the beams were aimed at the lesions. The lights were covered with a magenta colored filter (Rosculus #46 Magenta, 6% transmission, Rosco Laboratories, Stanford, Connecticut) to decrease the influence of the light on potential predator behavior. A magenta colored filter was selected based on the observation that artificial red light (> 680 nm wavelength) has little effect on fish behavior versus white light [28], [29]. No fish were observed to be attracted to the red lights. The camera was installed at 14:00 hours on 2 October 2010 and was removed at 12:00 on 3 October 2010. The video was reviewed for the interaction of any potential coral predators with the lesion. Start and end frames of the video were captured and analyzed using Image J for the progression of the lesions over the course of the video monitoring. The area of each lesion was measured in the recorded start and end frames and percent changes in lesion sizes between these two timepoints were calculated. Additionally, the linear progression rates of the lesions were measured with four distances as described above under *lesion progression rate calculation*.

#### Temperature measurements

Temperature data for the region were recorded by a continuous temperature logger deployed and maintained by the National Park Service at Haulover Bay, St. John, at 8 m depth [30]. Brewer's Bay is located approximately 30 km west of Haulover Bay and the corals observed in this study were located between 6 and 10 m in depth. Degree heating weeks (DHW) data were acquired from the records of NOAA Coral Reef Watch's near-real-time Satellite Bleaching Alert (SBA) System for the U.S. Virgin Islands; these data were collected within 50 km of the study site [31]. DHWs are a measure of accumulated heat stress based on an accumulation of “HotSpots”, which are instantaneous satellite-derived measurements of the occurrence and magnitude of thermal stress favorable for coral bleaching [32]. DHWs are expressed in °C-weeks and are calculated by NOAA Coral Reef Watch as 0.5 x Summation of previous 24 twice-weekly HotSpots [31]. The extent and magnitude of bleaching has been found to be more tightly associated with DHWs than with *in situ* temperature measurements [33], [34].

The study was conducted under permit # STT-004-11 issued by the U.S. Virgin Islands Department of Planning and Natural Resources to M. Brandt and T. Smith.

### Data analyses

#### Analysis of temporal patterns of bleaching and disease

Bleaching prevalence (% of the population affected by any level of bleaching) and disease prevalence (% of the population affected by disease) were calculated per transect. When analyzed for changes through time, bleaching and disease prevalence values did not meet the assumptions of a parametric Repeated-Measures Analysis of Variance (RM-ANOVA) test, even with transformation. Bleaching extent values, measured for each colony, also did not meet the assumptions of an RM-ANOVA. Therefore, to determine significant groups and temporal trends in these metrics, non-parametric Kruskal-Wallis tests and post-hoc pair-wise tests were used and adjusted with Bonferroni corrections.

#### Analysis of interactions among bleaching, disease, and fragmentation

To test our hypothesis that diseased and damaged corals would be more extensively bleached, a two-way Analysis of Covariance (ANCOVA) was performed with the bleaching extent of each colony as the response variable, and the colony's disease status (affected or unaffected) and fragmentation status (attached or unattached) as predictor variables. The size of a colony is known to affect bleaching extent [35], and was therefore included as a covariate.

To test the hypothesis that disease would occur more frequently on damaged corals, disease prevalences of attached and unattached colonies were compared. For this analysis, the % of attached colonies that were diseased was calculated for all transects and compared with the same metric for unattached colonies using a two sample t-test.

To determine whether the presence of disease on fragments was related to the substrate on which the fragment was found (unconsolidated sand or consolidated reef substrate), a contingency table analysis was applied to counts of colonies conforming to the following four combinations of conditions: 1) diseased/consolidated, 2) diseased/unconsolidated, 3) not diseased/consolidated, and 4) not diseased/unconsolidated. For this analysis, data from the first three surveys were also combined.

#### Analysis of interactions among disease and potential vectors

To determine whether disease was related to the presence of potential animal vectors of coral disease, contingency table analyses were applied to counts of colonies conforming to the following four combinations: 1) diseased/animal present, 2) diseased/animal absent, 3) not diseased/animal present, 4) not diseased/animal absent. In these analyses, data from the first three surveys were also combined and used with the exception of data from one observer who did not record interactions of colonies with any potential vector.

#### Analysis of spatial data

The mean density of diseased colonies in radial transects within five 1-m distance categories (0-1 m, 1–2 m, 2–3 m, 3–4 m and 4–5 m) was calculated and normalized to the area that each distance category represented. To determine whether diseased colonies were clustered, the distribution of the mean density of diseased colonies among the distance categories was then compared between disease-centered and unaffected-centered transects using a chi-square goodness of fit test. All statistical analyses were performed in JMP v. 9 (SAS Inc.).

## Results

**Figure 1:**
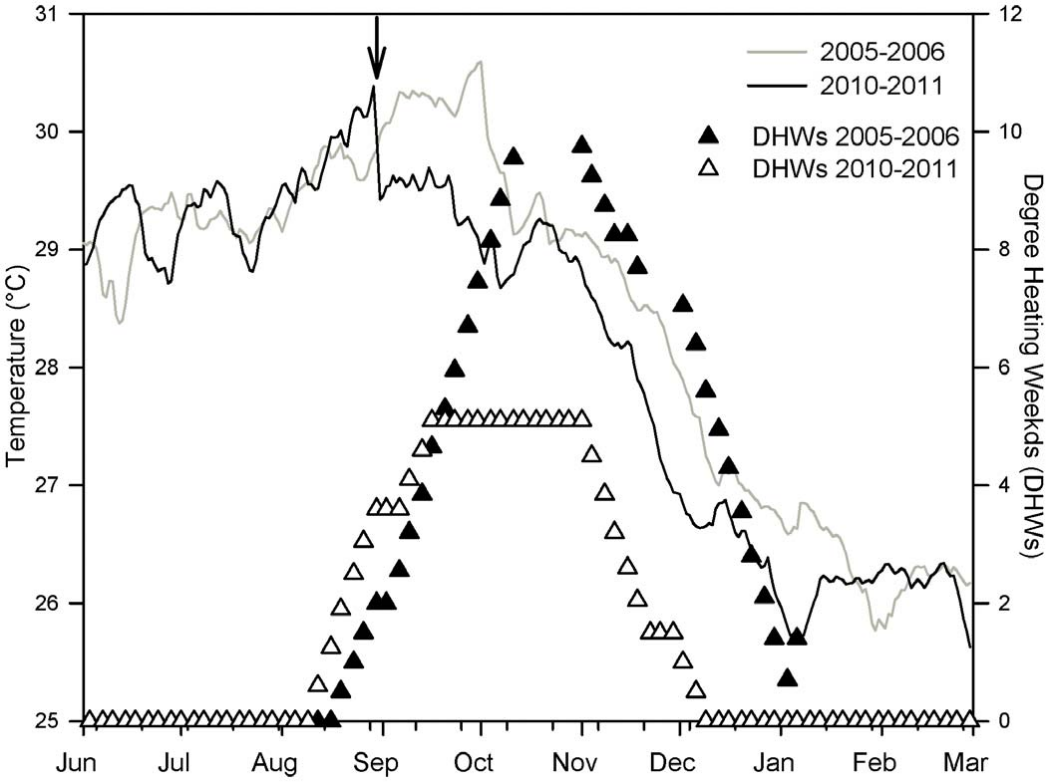
Temperature records. Temperature recorded *in situ* by the National Park Service and Degree Heating Weeks calculated by NOAA's Coral Reef Watch for the US Virgin Islands region during the study period in 2010 – 2011 as compared with the same time period spanning the 2005 mass bleaching event. Arrow indicates the date of passage of Hurricane Earl in 2010.

**Figure 2:**
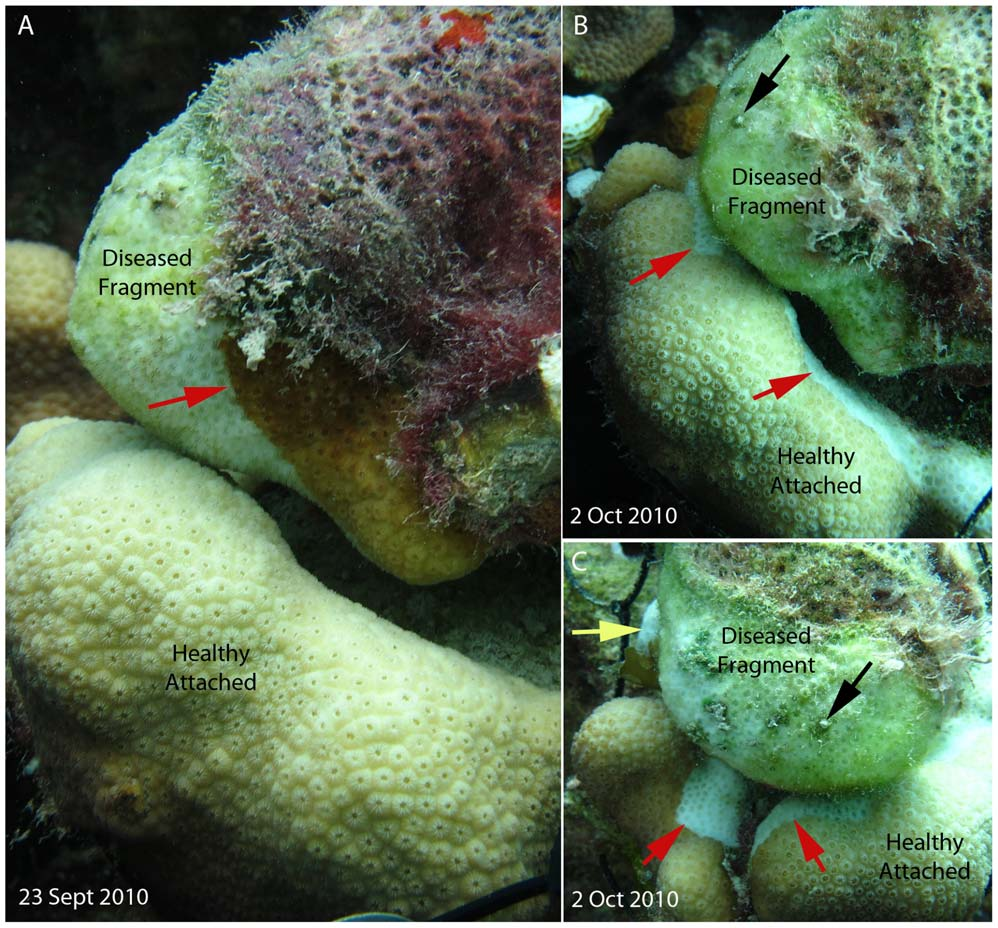
Example of disease transmission. A) Experimental set up of a diseased fragment attached to an unaffected colony. Red arrow indicates active advancing lesion. B) The same experimental setup three weeks later; the diseased fragment has experienced total mortality. Red arrows indicate where a new lesion has initiated on a previously unaffected colony. C) Second view of new lesions on the previously unaffected colony. Black arrows indicate identical spot in B, red arrows as in B, and the yellow arrow indicates a small colony of *Agaricia agaricites* that also was recently denuded of living tissue (See Supporting Information Figure S1).

In 2010, ocean temperatures in the U.S. Virgin Islands were following a warmer trajectory (Figure 1 black line) than had been observed in 2005 (Figure 1 grey line), a year in which an unprecedented mass bleaching event occurred in the territory [36]. By mid-August 2010, temperatures were above the bleaching threshold and degree heating weeks showed an accumulation of thermal stress in the USVI region (Figure 1 open triangles). High levels of bleaching were beginning to be observed at sites around the territory (T. Smith and M. Brandt pers. obs.). Hurricane Earl passed the territory on August 30 as a category 2 storm with sustained winds of 46 knots from the west-south west over four hours, contrary to normal wind direction and directly into Brewers Bay (weatherflow.com). This disturbance resulted in damage to and fragmentation of coral colonies on reefs most exposed to storm induced wave action. A decrease in temperatures and a slowing of thermal accumulation (Figure 1) also was associated with storm activity, an example of protective storm cooling [27]. In a survey of Brewer's Bay immediately following the passage of Hurricane Earl, an unusually high level (up to 8% prevalence) of white plague-like VI-MRTL was observed affecting corals, particularly colonies of the mounding, dominant reef-builder, *Montastraea annularis*. Lesions of this disease were identified as areas of skeleton recently denuded of tissue with little to no algal colonization (Figure 2). All observed lesions were located basally or peripherally on colonies and were multifocal to coalescing with distinct undulating margins.

### Temporal patterns of bleaching and disease

**Figure 3:**
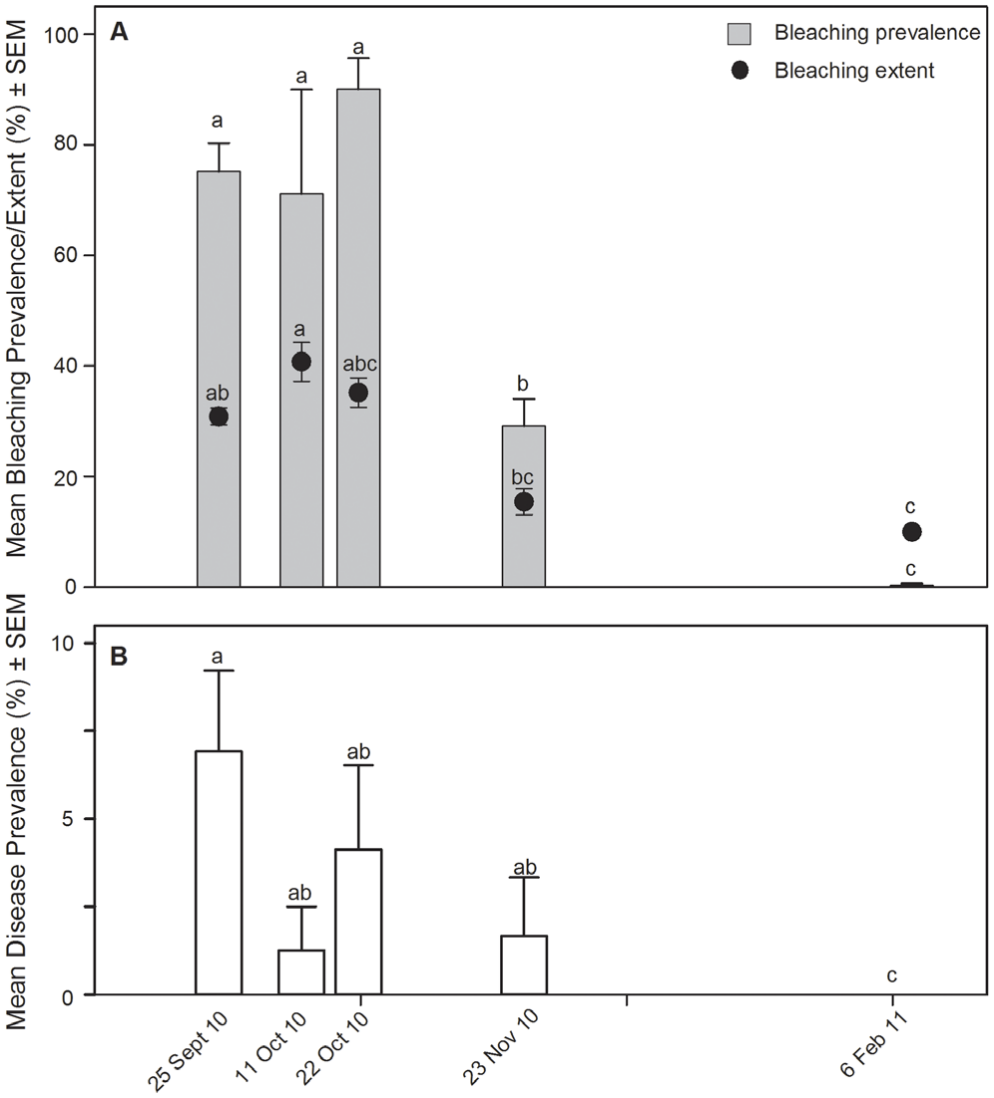
Bleaching and disease dynamics through time. A) Mean bleaching prevalence (% of population affected) and mean bleaching extent (% of colony area affected) at each survey point during the monitoring period (Sep 2010 – Feb 2011). Bleaching prevalence was calculated per transect; therefore sample sizes (# of transects) were, in chronological order, 6, 3, 3, 3 and 7. Bleaching extent was evaluated per each colony assessed; therefore sample sizes (# of colonies) were, in chronological order, 288, 84, 104, 55, and 172. B) Mean disease prevalence at each survey point during the monitoring period. Disease prevalence was calculated per transect; therefore sample sizes (# of transects) were as in A for bleaching prevalence. Letters in both graphs indicate significant groups (p < 0.05) as determined by post-hoc tests after significant Kruskal-Wallis tests.

Bleaching prevalence, bleaching extent and disease prevalence all significantly changed through time (KW tests: χ^2^  =  16.7, p  =  0.0022; χ^2^  =  18.1, p  =  0.023; χ^2^  =  11.3, p  =  0.0012, respectively). Bleaching prevalence and extent were high in September and October, but dropped by the time the site was surveyed in November (Figure 3A). Disease prevalence was as high as 6% in September and did not significantly decline until February (Figure 3B). When the site was visited in February, disease was not observed on any surveyed colonies and only one of the assessed colonies showed signs of bleaching (Figure 3).

### Abundance and distribution of coral fragments

Ninety-nine out of 450 (22%) *M. annularis* colonies surveyed within transects during the bleaching event (Sept – Oct) were not attached to the substrate. A little over half (55%) of these fragments were lying on unconsolidated substrate (sand) versus consolidated (hard) substrate.

### Interactions among bleaching, disease, fragmentation and substrate

**Figure 4:**
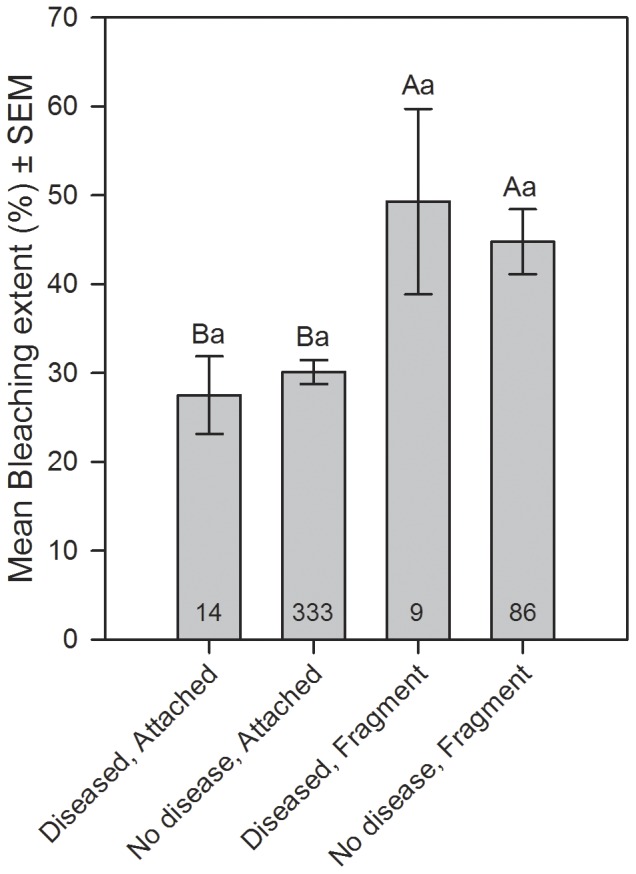
Interactions among bleaching, disease and fragmentation. Mean bleaching extent (% of colony affected) for categories of colonies surveyed including: 1) Diseased and structurally intact where no breakage was noted (“attached”), 2) unaffected by disease and attached, 3) diseased and no longer attached to the substrate with evidence of fresh breakage (“fragment”), and 4) unaffected by disease and fragment. Data were combined from the first three sampling periods during the peak of bleaching. Letters indicate significance (p < 0.05) as determined by a two-way ANCOVA for the effect of fragmentation (capital letters) and disease status (lowercase letters). Numbers at base of bars indicate the sample size (# of colonies).

The extent of bleaching on colonies was significantly higher on fragments than on attached colonies (ANCOVA: F  =  5.68, p < 0.05), but no difference in bleaching extent occurred on diseased versus unaffected colonies (F  =  0.53, p  =  0.47; Interaction: F  =  3.07, p  =  0.08; Figure 4).

**Figure 5:**
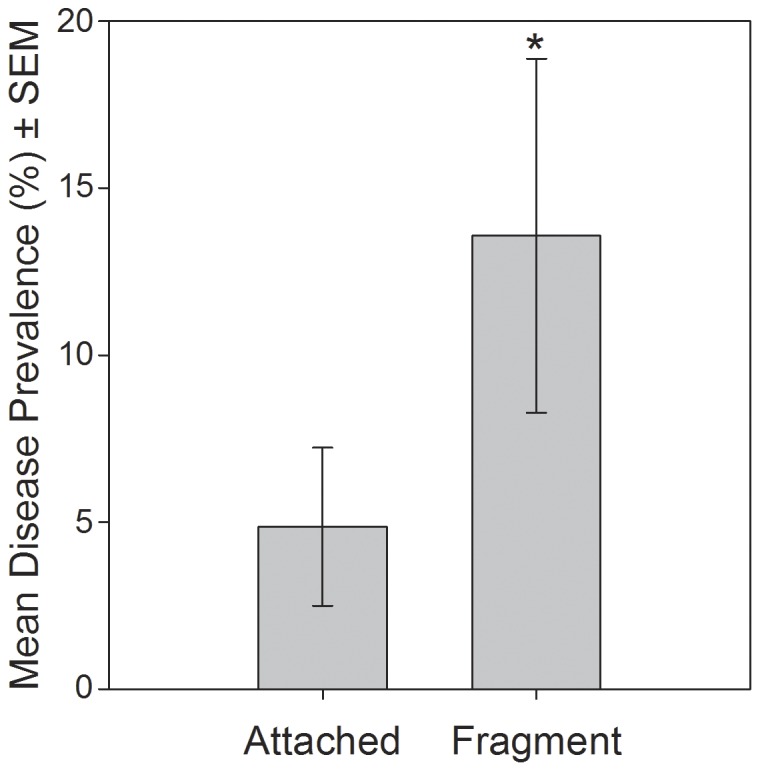
Disease prevalence and fragmentation. Mean disease prevalence on colonies that were structurally intact where no breakage was noted (“attached”) versus colony fragments that were no longer attached to the substrate and showed evidence of fresh breakage (“fragment”). Sample size was 12 for each category (# of transects assessed in the first three sample periods). Asterisk indicates significant difference as determined by a *t* test.

When tested independently of bleaching, disease prevalence was significantly higher on fragments versus attached colonies (*t*  =  2.31, p < 0.05) (Figure 5). When fragments were analyzed separately, disease occurred exclusively on fragments found on unconsolidated substrate while fragments unaffected by disease were found to be evenly distributed between both consolidated and unconsolidated substrate (Table 1, H_0_  =  1). The contingency table analysis of these frequencies determined that disease was statistically dependent upon whether the fragment was lying on sand or hard substrate (Table 1, H_0_  =  1).

**Table 1:** Tests of independence among coral categories.

H_0_:	Category	Diseased	Unaffected	Statistical Result
1	Consolidated	0 (5)	45 (40)	L ratio = 13.1, p < 0.001
	Unconsolidated	10 (5)	44 (49)	
2	Damselfish present	4 (5)	115 (114)	L ratio = 0.617, p = 0.43
	Damselfish absent	16 (15)	299 (300)	

Observed frequencies of coral conditions used in contingency table analyses with their expected frequencies in parentheses for tests of the null hypotheses: 1) Disease on coral fragments is independent of the substrate type (N  =  99)*, and 2) Disease is independent of the presence of the damselfish *Stegastes planifrons* (N  =  434).

* Only fragments were used in the analysis of the relationship between substrate type and disease.

### Interactions among disease and potential vectors

Known animal vectors of coral disease that were found on corals in this study included the polychaete *Hermodice carunculata*, the mollusk *Coralliophila abbreviata*, and the teleost *Stegastes planifrons*. *Hermodice carunculata* was observed on only one colony, and that colony was unaffected by disease. *Coralliophila abbreviata* was observed on 1.4% of colonies, but was not observed on any diseased coral. The damselfish *S. planifrons* was observed in association with 27% of colonies. When separated by disease status, *S. planifrons* was observed on 20% of diseased colonies and 28% of colonies unaffected by disease. No statistical relationship between the presence of this fish and disease was found (Table 1, H_0_  =  2). Macroalgae in direct contact with living coral tissue included *Dictyota* spp. and *Halimeda* spp. *Halimeda* spp. were found in contact with the living tissue of a colony unaffected by disease once, while *Dictyota* spp. were found in contact with the living tissue of 19 colonies, only one of which was diseased.

### Spatial data

**Figure 6:**
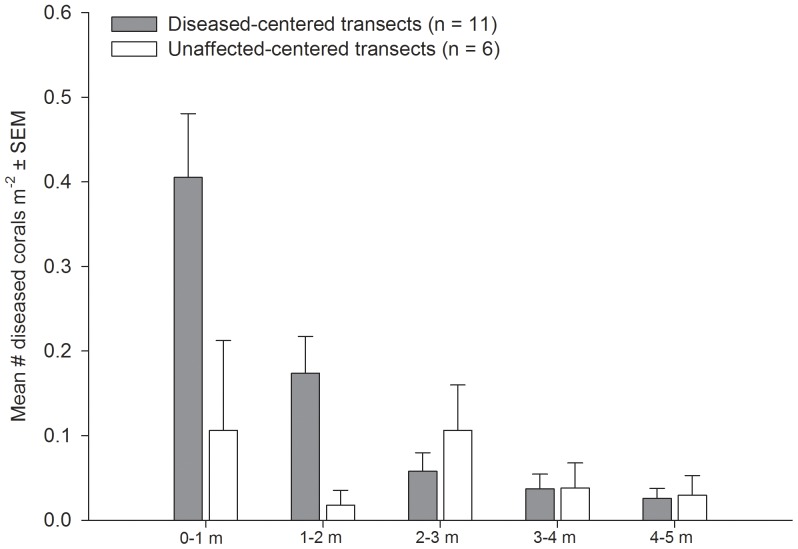
Spatial distribution of diseased colonies. Mean density of diseased colonies in 1 m distance categories surrounding randomly selected diseased and unaffected colonies.

The distribution of diseased corals in radial disease-centered transects was significantly different from radial unaffected-centered transects (Goodness of fit test: χ^2^  =  10.3, p < 0.05). In radial disease-centered transects the density of other diseased colonies tended to be higher in the closest distance categories, while in radial unaffected-centered transects diseased colony density was approximately equal across distance categories (Figure 6). The majority (111 of 119 colonies, 93%) of diseased colonies surveyed in radial transects were *M. annularis*, however, 3 of the 11 diseased-centered transects contained diseased colonies of multiple species. These other species included: *Diploria labyrinthiformis*, *D. strigosa*, *M. faveolata*, *Colpophyllia natans*, and *Eusmilia fastigiata*.

### Lesion progression rates

The overall mean linear progression rate of lesions was 0.23 cm/day ± 0.12 (SD). Minimum and maximum rates were 0.05 cm/day and 0.55/day cm, respectively.

### Transmission experiments

Two of 20 (10%) diseased pairs showed transmission of disease signs from diseased to unaffected colonies within 2 months (Figure 2). In one of these pairs, disease spread was limited to the colony in direct tissue contact with the diseased fragment; no other colonies were affected by disease in the surrounding area over the course of the experiment. In the other of these pairs, disease was transmitted from a fragment to an attached experimental coral, and also caused total mortality on a small (∼2 cm) colony of *Agaricia agaricites* that was located on a dead area of the originally unaffected experimental coral (Figure 2C, yellow arrow). This *A. agaricites* colony (Figure S1) was not in direct tissue-to-tissue contact but was located 10 cm away from the disease lesion that appeared on the attached experimental coral. Control pairs showed no appearance of disease signs or lesions of any kind.

### Video record of lesions

**Figure 7:**
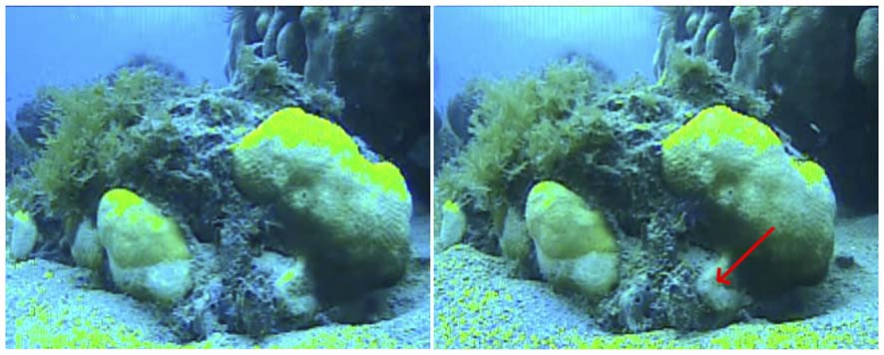
Still frames from the start (left) and end (right) of a continuous 22-hour video focused on two disease lesions. Red arrow indicates area of tissue loss.

A 22-hour video of a coral lesion showed that the lesion area increased by 2.4% (from 16.92 cm^2^ to 17.34 cm^2^, Figure 7); no visible interactions with a coral predator occurred. The linear progression rate of the lesion was 0.34 cm/day ± 0.06 (SD). This rate was within the variance (mean 0.23 cm/day ± 0.12 SD) recorded for the corals photographed for the *lesion progression rate calculation*.

## Discussion

VI-MRTL lesion progression rates were most consistent with white plague type 1 (reported as a maximum of 3 mm/day by Dustan [13]). However, original descriptions of lesion progression for types 2 and 3 were reported in the literature as maximum rates (not mean rates), precluding comparisons with VI-MRTL lesions. Overall, VI-MRTL seemed most closely aligned with descriptions for type 1, yet VI-MRTL affected *D. strigosa* and *E. fastigiata* corals, and white plague type 1 has not previously been observed on these corals [9].

The prevalence of VI-MRTL was associated with colony fragmentation and physical contact with sediment. Direct transmission of VI-MRTL via tissue-to-tissue contact also was demonstrated in 10% of experimental colony pairs and the clustered spatial distribution of the disease in radial transects suggests that transmission was occurring. We therefore propose that primary incidence of disease occurred on corals as a result of mechanical damage and exposure to sediment and secondary transmission also occurred, through some unidentified mechanism. Outbreaks of white diseases have been associated with mechanical sources of damage and stress in the past. On reefs of the Caribbean island of Navassa, storm related impacts may have contributed to high levels of a white plague-like disease observed one month following the passage of two hurricanes [23]. Similarly, an outbreak of a white plague-like disease occurred within weeks of the passage of a hurricane in Puerto Rico in 1996 [37]. In both studies, mechanical damage experienced by colonies was noted, although specific connections between damage and disease were not evaluated. In experimental studies, mechanical damage also has been suggested to play a role in the transmission of disease. For example, Nugues et al. [38] suggested that algal abrasion might have been the mechanism of pathogen invasion in their studies of the relationship between white plague type 2 disease and the calcareous green alga, *Halimeda opuntia*. In a separate study of the coral disease black band, disease transmission only occurred in the presence of a coral predator and predation scars were the sites of lesion development [39]. These studies suggest that the physical disruption of coral tissue may provide a primary site of invasion for disease pathogens. In the disease outbreak documented here, lacerations to coral tissue during hurricane-induced colony fragmentation and sediment scouring likely provided points of entry to the causative agent, which could potentially be a normal constituent of the coral surface mucous layer, sediments, or the water column.

The spatial distribution of VI-MRTL was clustered, indicating that some transmission was occurring [40]. Other studies have described clustered distributions for white disease affected colonies [41], [42], [43]. Among these studies, Brandt and McManus [43] implemented identical methods to those used here, and found clustering of putative white plague type 2 diseased colonies, particularly within the first meter of a diseased central colony, corroborating our results. In this study, we demonstrated direct transmission, albeit at a low rate (10%). Given the extreme difficulty of completely securing coral pairs in the field, the transmission rate reported here is likely conservative. Higher rates of transmission may be observed if further experimentation is conducted in a controlled laboratory setting; however, it is unlikely that tissue-to-tissue contact was the dominant transmission mechanism in this outbreak. Most colonies in Brewer's Bay are not in direct contact with other colonies, but transmission may have occurred via some other mechanism that operated over short distances. For example, the movement of infected fragments around the study site could have resulted in the transmission of disease. Further experimentation with infected fragments might elucidate this as a potential mechanism of disease transmission.

Our statistical analyses of associations between VI-MRTL and animals demonstrated to be vectors or potential vectors in other coral disease systems (i.e., *H. carunculata, C. abbreviata*, *S. planifrons*) did not reveal any suggestive patterns. The green macroalga species *Halimeda opuntia*, a known vector of white plague type 2 disease [38], also was not associated with diseased colonies and is rare in Brewer's Bay (T. Smith data unpublished). The brown macroalgae species group *Dictyota* was recently correlated with a white plague-like outbreak in the Western Atlantic [44] and associated with significant changes in the coral microbiome [45], [46]. *Dictyota* was abundant at the study site during the outbreak, but was observed to be in contact with only one diseased colony. In laboratory experiments with the white plague type 2 pathogen, water-borne transmission of disease signs was demonstrated [14]. In this study, water-borne transmission would have likely resulted in larger sized clusters or a random distribution of affected colonies, unlike what was observed. However, if the causative agent/agents had limited viability in the water this could have resulted in short-range transmission and the observed tightly clustered pattern of diseased corals. Further studies into the oceanographic processes and distribution of potential vectors in relationship to disease at the reef scale (< 1 km) would be helpful for understanding whether water motion or coral predators could have produced the spatial disease prevalence patterns reported here.

The possibility also exists that the clustered distribution of diseased colonies reflected the spatial distribution of genetically susceptible coral colonies. Corals are colonial organisms that depend partially on asexual reproduction; therefore the clumped nature of disease-affected colonies could have been the result of highly susceptible clones being distributed close to each other. However, disease clusters with multi-species membership also were observed, indicating disease colony clustering independent of genetic distribution. The patterns identified in these studies suggest that Caribbean white diseases are the result of an infectious and transmissible etiology, but only further experimentation in the field and under controlled laboratory conditions will allow us to determine the mechanism or mechanisms of their transmission.

Surprisingly, disease was not statistically associated with bleaching stress, although the peak in disease prevalence corresponded with a peak in bleaching prevalence. Other studies have found significant associations between bleaching and disease [21], including bleaching and white disease on *Montastraea* spp. [24]. Increased disease susceptibility associated with bleaching is potentially the result of the acute and chronic negative consequences to a coral's condition from prolonged bleaching. Bleaching can result in decreased growth [47], lowered energy reserves [48], and shifts in the surface mucosal microbial communities which under normal circumstances confer resistance to potential pathogens [49]. These negative effects from bleaching may decrease a coral's resistance to pathogens and result in higher incidence of disease associated with greater levels of bleaching. In Brandt and McManus [24], disease incidence (defined as the rate of new cases of disease) primarily occurred after bleaching had subsided and colonies that became affected by white plague experienced higher bleaching extents than colonies that did not become diseased. The authors suggested that this might have been due to a weakening of coral energy reserves and a loss of disease defenses. In our study, disease was recorded at the peak of bleaching and declined corresponding with declines in bleaching prevalence, but no statistical relationship existed between bleaching extent and disease status.

The discrepancy in temporal patterns and relationships between bleaching and disease between this study and Brandt and McManus [24] could be due to differences in etiologies (although gross sign descriptions were similar), host species (*M. annularis* versus *M. faveolata*), or methods; this study assessed random colonies on each visit, while Brandt and McManus [24] followed individual colonies through time. However, the timing of hurricane impacts, particularly increased turbidity and re-suspension of sediment, may have played a role in both instances. In Brandt and McManus [24], thermal stress, and consequently bleaching, declined in response to Hurricane Wilma, and disease incidence was highest immediately after the storm's passage. Little mechanical damage to coral colonies due to the passage of Hurricane Wilma was observed by the authors (Brandt pers. obs.), but re-suspension of sediments resulted in high turbidity [27]. Re-suspension of sediments and high turbidity also were observed in this study following the passage of Hurricane Earl. Both our site and the sites in Brandt and McManus [24] were shallow, located close to shore and were dominated by *Montastraea* spp. (e.g., *M. annularis* and *M. faveolata*).

No other monitored *Montastraea* spp.-dominated shallow embayments in the USVI had unusually high disease prevalence during the event reported here (T. Smith unpublished data, [50]), yet Brewer's Bay is situated in such a way (facing southwest) that it received a large amount of swell resulting in greater turbidity than at other sites. In this study, disease on fragments was associated with location on and contact with unconsolidated substrate. Therefore, contact with sediment may have played a significant role in the initiation of disease signs. It is difficult to confirm that changes in water quality played a role in the disease outbreaks recorded here and in Brandt and McManus [24], however, since turbidity was not measured in either study. Additionally, outbreaks of a similar white plague-like disease in the USVI following the 2005 mass bleaching event [22] were not associated with storm passage or storm-related sediment suspension as no hurricanes impacted the region during that time [27]. However, the possibility remains that increased turbidity due to the passage of storms may have resulted in water quality changes that decreased disease resistance in coral hosts or increased pathogen abundance, virulence, or interaction with hosts.

The group of rapid tissue loss diseases referred to as white diseases is an increasingly important source of coral mortality on reefs, particularly in the Caribbean. In this study we have identified mechanical disturbance and exposure to sediment as probable factors involved in the primary incidence of a white plague-like rapid tissue loss disease with a unique suite of epidemiological characteristics. We also have identified that this disease likely underwent secondary transmission within the studied population, although the mechanism for secondary transmission remains unknown. More research is needed into the epidemiological characteristics of white plague-like rapid tissue loss diseases. These types of studies performed in conjunction with investigations of rapid tissue loss disease etiological agents and their origins will enable the scientific community to ultimately predict and possibly prevent future outbreaks.

## Supporting Information

Figure S1 **Additional view of experimental set up presented in** **Figure 2B and C** **taken 2 October 2010.** This view shows more clearly the small colony of *Agaricia agaricites* recently denuded of living tissue indicated by a yellow arrow in this view and in Figure 2C. Red arrow indicates active advancing lesion; the same lesion is indicated by the left red arrow in Figure 2C.
(TIF)
