# Supplementary figures and images for: Disturbance Driven Colony Fragmentation as a Driver of a Coral Disease Outbreak
Source: PLoS One. 2013 Feb 20;8(2):e57164. doi: 10.1371/journal.pone.0057164 (PMC3577774; doi:10.1371/journal.pone.0057164)

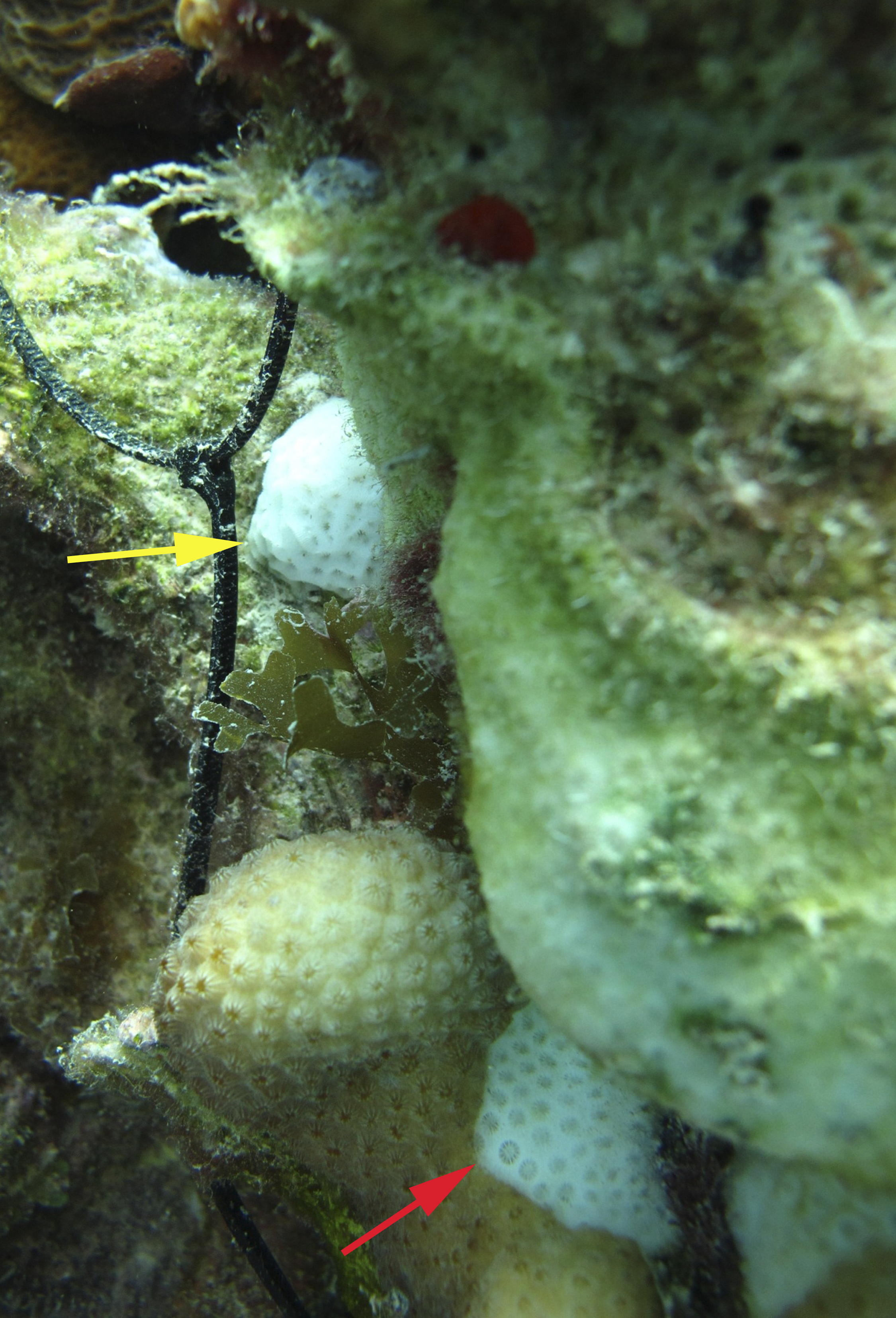

Supplement: Figure S1 — Additional view of experimental set up presented in Figure 2B and C taken 2 October 2010. This view shows more clearly the small colony of Agaricia agaricites recently denuded of living tissue indicated by a yellow arrow in this view and in Figure 2C. Red arrow indicates active advancing lesion; the same lesion is indicated by the left red arrow in Figure 2C. (TIF) [file pone.0057164.s001.tif]
